# Description of the Method for Evaluating Digital Endpoints in Alzheimer Disease Study: Protocol for an Exploratory, Cross-sectional Study

**DOI:** 10.2196/35442

**Published:** 2022-08-10

**Authors:** Jelena Curcic, Vanessa Vallejo, Jennifer Sorinas, Oleksandr Sverdlov, Jens Praestgaard, Mateusz Piksa, Mark Deurinck, Gul Erdemli, Maximilian Bügler, Ioannis Tarnanas, Nick Taptiklis, Francesca Cormack, Rebekka Anker, Fabien Massé, William Souillard-Mandar, Nathan Intrator, Lior Molcho, Erica Madero, Nicholas Bott, Mieko Chambers, Josef Tamory, Matias Shulz, Gerardo Fernandez, William Simpson, Jessica Robin, Jón G Snædal, Jang-Ho Cha, Kristin Hannesdottir

**Affiliations:** 1 Novartis Institutes for Biomedical Research Basel Switzerland; 2 Novartis Pharmaceuticals Corporation East Hanover, NJ United States; 3 Novartis Institutes for Biomedical Research Cambridge, MA United States; 4 Altoida Inc Washington, DC United States; 5 Global Brain Health Institute Trinity College Dublin Ireland; 6 Cambridge Cognition Ltd Cambridge United Kingdom; 7 MindMaze SA Lausanne Switzerland; 8 Linus Health Boston, MA United States; 9 Massachusetts Institute of Technology Cambridge, MA United States; 10 Neurosteer Inc New York, NY United States; 11 Neurotrack Technologies Inc Redwood City, CA United States; 12 Department of Medicine School of Medicine Stanford University Stanford, CA United States; 13 Neurovision Imaging Inc Sacramento, CA United States; 14 ViewMind Inc New York, NY United States; 15 Winterlight Labs Toronto, ON Canada; 16 Memory Clinic Landspitali Reykjavik Iceland

**Keywords:** digital endpoints, cognition, Alzheimer disease, brain amyloid, methodology study, clinical trial design, mobile phone

## Abstract

**Background:**

More sensitive and less burdensome efficacy end points are urgently needed to improve the effectiveness of clinical drug development for Alzheimer disease (AD). Although conventional end points lack sensitivity, digital technologies hold promise for amplifying the detection of treatment signals and capturing cognitive anomalies at earlier disease stages. Using digital technologies and combining several test modalities allow for the collection of richer information about cognitive and functional status, which is not ascertainable via conventional paper-and-pencil tests.

**Objective:**

This study aimed to assess the psychometric properties, operational feasibility, and patient acceptance of 10 promising technologies that are to be used as efficacy end points to measure cognition in future clinical drug trials.

**Methods:**

The Method for Evaluating Digital Endpoints in Alzheimer Disease study is an exploratory, cross-sectional, noninterventional study that will evaluate 10 digital technologies’ ability to accurately classify participants into 4 cohorts according to the severity of cognitive impairment and dementia. Moreover, this study will assess the psychometric properties of each of the tested digital technologies, including the acceptable range to assess ceiling and floor effects, concurrent validity to correlate digital outcome measures to traditional paper-and-pencil tests in AD, reliability to compare test and retest, and responsiveness to evaluate the sensitivity to change in a mild cognitive challenge model. This study included 50 eligible male and female participants (aged between 60 and 80 years), of whom 13 (26%) were amyloid-negative, cognitively healthy participants (controls); 12 (24%) were amyloid-positive, cognitively healthy participants (presymptomatic); 13 (26%) had mild cognitive impairment (predementia); and 12 (24%) had mild AD (mild dementia). This study involved 4 in-clinic visits. During the initial visit, all participants completed all conventional paper-and-pencil assessments. During the following 3 visits, the participants underwent a series of novel digital assessments.

**Results:**

Participant recruitment and data collection began in June 2020 and continued until June 2021. Hence, the data collection occurred during the COVID-19 pandemic (SARS-CoV-2 virus pandemic). Data were successfully collected from all digital technologies to evaluate statistical and operational performance and patient acceptance. This paper reports the baseline demographics and characteristics of the population studied as well as the study's progress during the pandemic.

**Conclusions:**

This study was designed to generate feasibility insights and validation data to help advance novel digital technologies in clinical drug development. The learnings from this study will help guide future methods for assessing novel digital technologies and inform clinical drug trials in early AD, aiming to enhance clinical end point strategies with digital technologies.

**International Registered Report Identifier (IRRID):**

DERR1-10.2196/35442

## Introduction

### Background

Alzheimer disease (AD) is a progressive and terminal illness and the most common form of dementia, with a rapidly growing societal and economic burden [1]. Patients with AD present with gradual and wide-ranging cognitive and functional impairments, as well as loss of motivation, social withdrawal, and other neuropsychiatric challenges [2,3]. The standard of care for AD is based on providing patients with symptomatic relief; however, these therapies are unsatisfactory and provide limited efficacy [4]. In recent years, drug development has largely focused on disease-modifying treatments to stop, slow, or prevent disease progression. However, because of the high clinical trial failure rate, AD remains the illness with the highest unmet medical need in neuroscience [5]. This high failure rate might be because of the multifactorial etiology of AD and the large diversity of the clinical manifestations in each patient. However, these failures may also be partially because of weak efficacy end points that cannot reliably and accurately demonstrate drug treatment effects across heterogeneous patient populations.

Currently, the standard method for assessing cognition in clinical drug trials is modeled on traditional neuropsychological paper-and-pencil assessments that tend not to be optimal for frequent monitoring of drug treatment effects, primarily because of practice effects, high variability and burden, single–time point administrations, and poor psychometric properties such as ceiling and floor effects. Ceiling and floor effects pose critical risks to the accurate monitoring of immediate symptomatic drug treatment enhancements of cognition and longitudinal disease-modifying effects on disease progression. Moreover, the information obtained from traditional clinical trial end points is often reduced to a single total score, thereby potentially losing important clinical insights into drug treatment effects. In reality, as with most cognitive functions required in daily life, solving these tests involves the orchestration of several cognitive domains operating together. In clinical trial settings, single–time point paper-and-pencil tests often provide limited and inaccurate information about central nervous system functioning and have poor sensitivity to drug treatment effects. For someone who is not a trained expert, the paper-and-pencil tests can be burdensome and complex to administer, often resulting in rater errors, high variability, and small drug treatment effect sizes. Poor sensitivity to changes and limitations of conventional end points often leads to large, lengthy, and costly trials. Finally, many of these paper-and-pencil tests are quite subjective and, as a result, may not reflect the reality of the symptoms, for example, because of expectations of drug treatment effects and anosognosia [6]. Taken together, poor efficacy end points pose serious risks to neuroscience drug development.

There is an urgent need for improved clinical trial efficacy end points, and new assessments using novel digital technologies are rapidly emerging. For instance, using sensor technology to collect physiological data during cognitive assessments allows for a richer evaluation of central nervous system functioning that cannot be obtained by means of conventional paper-and-pencil administration only. The combination of several sensors measuring motion, voice, and brain activity within different test modalities allows for a high resolution of patient symptoms. Gamification via augmented reality (AR) technology is a novel and promising approach that could offer ecological validity to cognitive and functional assessments [7]. Moreover, digital technologies allow for the implementation of an adaptive level of difficulty to avoid ceiling and floor effects [8], which are important psychometric limitations of many conventional paper-and-pencil tests. Although novel sensor technologies hold promise for improved clinical trials in AD, it remains unclear how best to evaluate these technologies or how to use them to derive efficacy end points that can more effectively detect drug treatment effects. With regard to the development of improved assessment tools, the technology, operational feasibility, and usability need to be carefully considered to suit the perceptual and interaction needs of clinical trial participants with cognitive impairments.

**Figure 1:**
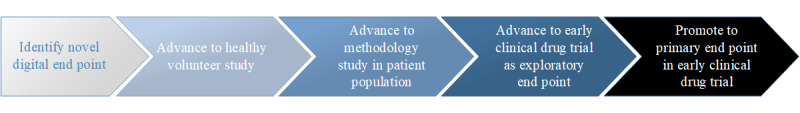
A road map to advance digital end points within the drug development process.

Hannesdottir et al [9] previously proposed a road map to advance novel digital end points within the early drug development process (Figure 1).

The initial step once a promising digital end point has been identified involves technical verification in healthy controls to determine whether the digital end point is ready for testing in the target patient population. The next step of the road map involves running a digital end point methodology study to provide technology and operational feasibility, psychometric properties, and patient acceptance in the target population. Digital technologies that successfully meet predefined success criteria based on a previously developed scoring system [9] can then be advanced to the next step, which involves studying the digital technology in a phase 2 clinical drug trial as an exploratory end point. In the phase 2 trial, the sensitivity of the digital end point to drug treatment effects can be compared head to head with conventional paper-and-pencil end points. If the digital end point is considered clinically meaningful and produces less variability and greater drug signal detection than the conventional end points, the digital end point may eventually advance to the final step of the road map and be used as a primary end point to run smaller and shorter phase 2 trials in the same target population.

### Objectives

In light of the urgent need for improved efficacy end points for clinical drug trials and the rapid surge of promising digital technologies, the aim of the Method for Evaluating Digital Endpoints in Alzheimer Disease (MEDIA) study is to assess the psychometric properties, operational feasibility, and patient acceptance of 10 promising technologies for measuring cognition to be used as efficacy end points in future clinical drug trials. Each of the novel digital technologies will be compared against established paper-and-pencil end points in their ability to accurately classify participants into 4 cohorts of cognitive impairment and dementia severity. This paper describes the MEDIA study protocol and participant demographics, the role this study plays in the drug development process, and some of the limitations of the study.

## Methods

### Study Design

**Figure 2:**
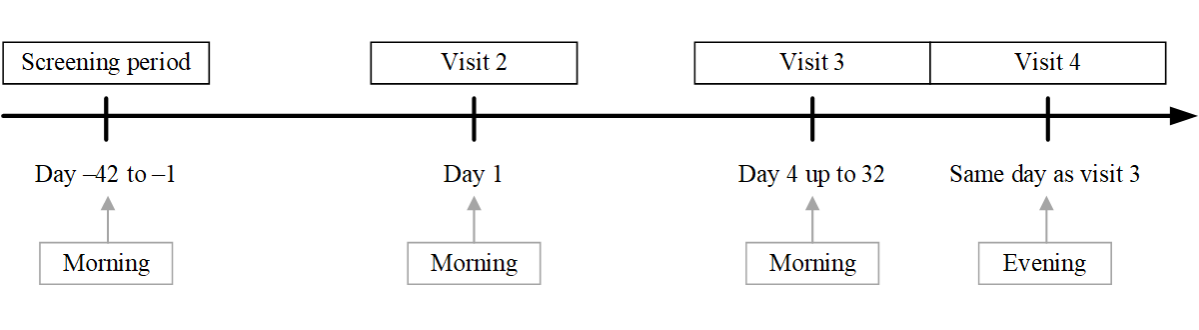
Method for Evaluating Digital Endpoints in Alzheimer Disease study design.

This is a cross-sectional, noninterventional study conducted at the Memory Clinic at Landspítali University Hospital in Iceland. The total study duration (including a screening period of up to 42 days) was a maximum of 74 days, allowing for scheduling flexibility but avoiding the effects of disease progression [10]. Assessments were completed at visit 1 (ie, screening visit, occurring 1-42 days before day 1), visit 2 (day 1), visit 3 (day 4 to day 32, morning), and visit 4 (evening of the same day as visit 3; Figure 2).

**Figure 3:**
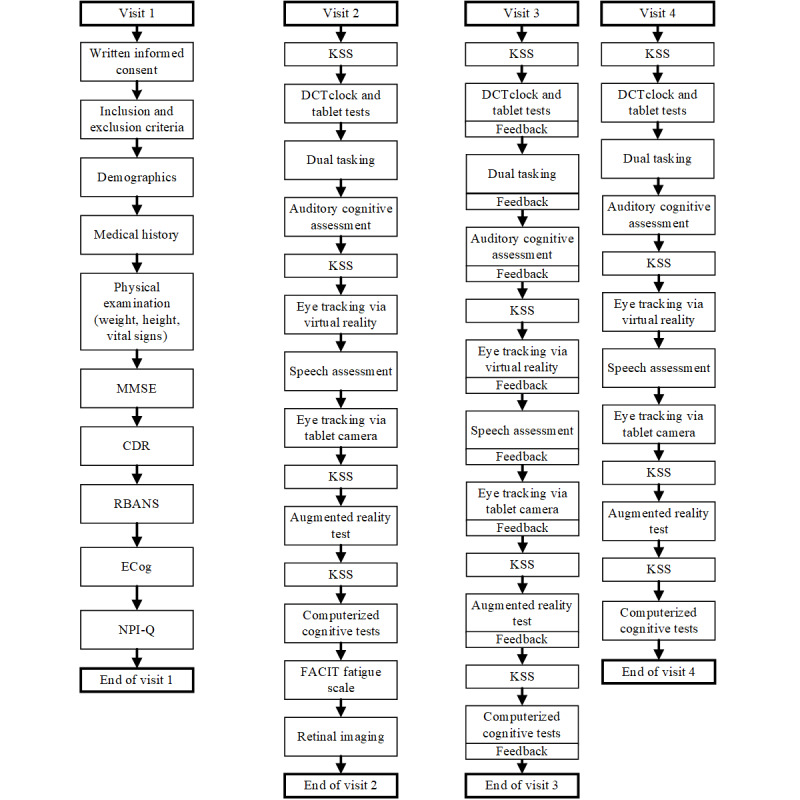
Order of assessments at each visit of the MEDIA study. CDR: Clinical Dementia Rating scale; ECog: Everyday Cognition scale; FACIT: Functional Assessment of Chronic Illness Therapy; KSS: Karolinska Sleepiness Scale; MEDIA: Method for Evaluating Digital Endpoints in Alzheimer Disease; MMSE: Mini-Mental State Examination; NPI-Q: Neuropsychiatric Inventory–Questionnaire; RBANS: Repeatable Battery for the Assessment of Neuropsychological Status.

At visits 1, 2, and 3, assessments were conducted in the morning to avoid the effect of circadian fluctuation in AD [11]. At visit 4, a benign cognitive challenge model was implemented to assess the sensitivity of digital end points to change. Fatigue and sleep deprivation have been shown to affect performance across a wide range of cognitive domains [12,13]. To produce cognitive fatigue, all assessments during visit 4 were conducted in the evening. No napping was allowed before the evening assessments, and no caffeine or other stimulants were allowed after noon. The order of assessments at each visit was predefined, as illustrated in Figure 3.

### Participants

The study was conducted at an academic Memory Clinic in the Geriatric Department of Landspítali University Hospital in Reykjavik, Iceland. Participants were grouped into 4 cohorts derived from 2 sources. The 2 cohorts comprised cognitively healthy amyloid-negative (controls; cohort 1) and cognitively healthy amyloid-positive (presymptomatic; cohort 2) male and female participants. These participants had been investigated using either cerebrospinal fluid (CSF) analysis or amyloid positron emission tomography (PET) up to 2 years earlier. The other 2 cohorts comprised male and female individuals who had been referred to the Memory Clinic by their primary health care physician and had either been diagnosed with mild cognitive impairment (MCI; predementia; cohort 3) or mild AD (mild dementia; cohort 4) at the Memory Clinic. The participants were aged between 60 and 80 years. The clinical diagnosis of MCI and mild AD was made according to the National Institute on Aging and Alzheimer Association criteria [14]. A total of 53 participants were enrolled in the study, of whom 3 (6%) failed the screening criteria (because of the Mini-Mental State Examination score being <20), resulting in a total of 50 (94%) participants who were included in the study: 13 (26%) were controls, 12 (24%) were presymptomatic, 13 (26%) were predementia, and 12 (24%) had mild dementia. Of the 50 participants, 4 (8%) discontinued the study after finalizing visit 2; hence, 46 (92%) participants completed all study visits: 12 (26%) were controls, 12 (26%) were presymptomatic, 11 (24%) were predementia, and 11 (24%) had mild dementia. The data of all 50 participants were used for further analysis where applicable; that is, for all analyses performed on the data up to visit 3. The detailed inclusion and exclusion criteria are listed in Multimedia Appendix 1, and protocol deviations are listed in Multimedia Appendix 2.

### Ethics Approval

Written informed consent was obtained from all participants. The study was approved by the National Bioethics Committee (reference number VSN-20-022) in Reykjavik, Iceland, and conducted in accordance with the National Bioethics Committee’s ethical standards and the latest version of the Declaration of Helsinki.

### Conventional Paper-and-Pencil End Points

#### The Repeatable Battery for the Assessment of Neuropsychological Status

The Repeatable Battery for the Assessment of Neuropsychological Status (RBANS) [15] is a clinical tool specifically designed for both diagnostic purposes and for tracking changes in neurocognitive status over time. The RBANS was selected for the MEDIA study as this battery was designed to detect and characterize the earliest neurocognitive changes associated with dementia. RBANS scores have been reported to be correlated with cerebral amyloid in both cognitively normal individuals [16] and patients with MCI because of AD [17]. The RBANS takes 30 to 40 minutes to administer and generates age-adjusted index scores for 5 neurocognitive domains that are used to calculate a total scale index score (total possible range 40-160; a higher score indicates better cognitive function). It comprises the following domains with associated subtests used for index scores: (1) Immediate Memory (List Learning and Story Memory), (2) Visuospatial and Constructional (Figure Copy and Line Orientation), (3) Language (Picture Naming and Semantic Fluency), (4) Attention (Digit Span and Coding), and (5) Delayed Memory (List Recall, List Recognition, Story Memory, and Figure Recall).

#### Mini-Mental State Examination

The Mini-Mental State Examination is a brief, practical, clinician-reported outcome that examines cognitive status [18]. It evaluates orientation, memory, attention, concentration, naming, repetition, comprehension, and the ability to create a sentence and copy 2 intersecting pentagons. The test comprises 5 sections (orientation, registration, attention, recall, and language), with a total score ranging from 0 to 30. Higher scores indicate better cognitive function.

#### Clinical Dementia Rating Scale

The Clinical Dementia Rating scale (CDR) is a global measure of cognitive and functional performance and is widely used in clinical research on AD [19]. The scale assesses 6 domains: memory, orientation, judgment and problem-solving, community affairs, home and hobbies, and personal care. Each domain is assigned a score that can be summed to obtain the sum of boxes (CDR Sum of Boxes [CDR-SOB]) score. The necessary information for assessment is obtained through a semistructured interview with the participant and a reliable informant or collateral source (ie, study partner). Descriptive anchors are provided for each score, which guides the clinician in making appropriate ratings based on interview data and clinical judgment to evaluate the staging severity of dementia. Global CDR scores and CDR-SOB scores were also collected. Global CDR scores range from 0 to 3, with greater scores indicating greater disease severity. CDR-SOB scores range from 0 to 18, with greater scores indicating greater disease severity.

#### Neuropsychiatric Inventory–Questionnaire

The Neuropsychiatric Inventory (NPI) assesses a wide range of behaviors encountered in patients with dementia. The NPI-Questionnaire (NPI-Q) is a questionnaire (adapted from the NPI [20] and omitting the frequency rating) that is well-suited for use in general clinical practice settings [21]. In the NPI-Q, the study partner (informant) is asked whether the participant has experienced a variety of neuropsychiatric symptoms in the past month, which are then assessed in terms of severity on the same 3-point scale as in the original NPI (1=mild, 2=moderate, and 3=severe) using similar anchor points. The total NPI-Q severity score represents the sum of the individual symptom scores and ranges from 0 to 36. The total NPI-Q severity score and the individual symptom scores were recorded. Informant distress scores were not collected in this study.

#### Everyday Cognition Scale

The Everyday Cognition scale (ECog) measures cognitively relevant everyday abilities and comprises 39 items covering 6 cognitively relevant domains: Everyday Memory, Everyday Language, Everyday Visuospatial Abilities, Everyday Planning, Everyday Organization, and Everyday Divided Attention [22]. The questionnaire is a self-reported measure completed by both the participant (ECog-participant) and their study partner (ECog-informant). Within each domain, the ability to perform a specific task is rated on a 5-point scale ranging from (1) no difficulty, (2) mild difficulty, (3) moderate difficulty, (4) severe difficulty, or (5) unable to do. The total score for the 39 items ranges from 39 to 195, with greater scores indicating worse daily function. The 39-item data and total scores were collected. Details on study partner characteristics (relationship and frequency of interaction) are also captured on the ECog-informant.

#### The Functional Assessment of Chronic Illness Therapy–Fatigue scale

The Functional Assessment of Chronic Illness Therapy–Fatigue Scale is a short 13-item questionnaire that measures an individual’s level of fatigue during their usual daily activities over the past week. The level of fatigue is measured on a 4-point Likert scale ranging from 0 (very much fatigued) to 4 (not at all fatigued) [23].

#### Karolinska Sleepiness Scale

Participants completed the Karolinska Sleepiness Scale before starting the testing at each study visit to assess their level of sleepiness [24]. This scale was completed at 4 different time points during the testing at each study visit to assess the change in the sleepiness of each participant over the time they were tested. The Karolinska Sleepiness Scale is a 1-question scale that measures the level of sleepiness or alertness in 9 steps: *extremely alert*; *very alert*; *alert*; *rather alert*; *neither alert nor sleepy*; *some signs of sleepiness*; *sleepy, but no effort to keep awake*; *sleepy, some effort to keep awake*; and *very sleepy, great effort to keep awake, fighting sleep.*

### Digital End Points

#### Overview

**Figure 4:**
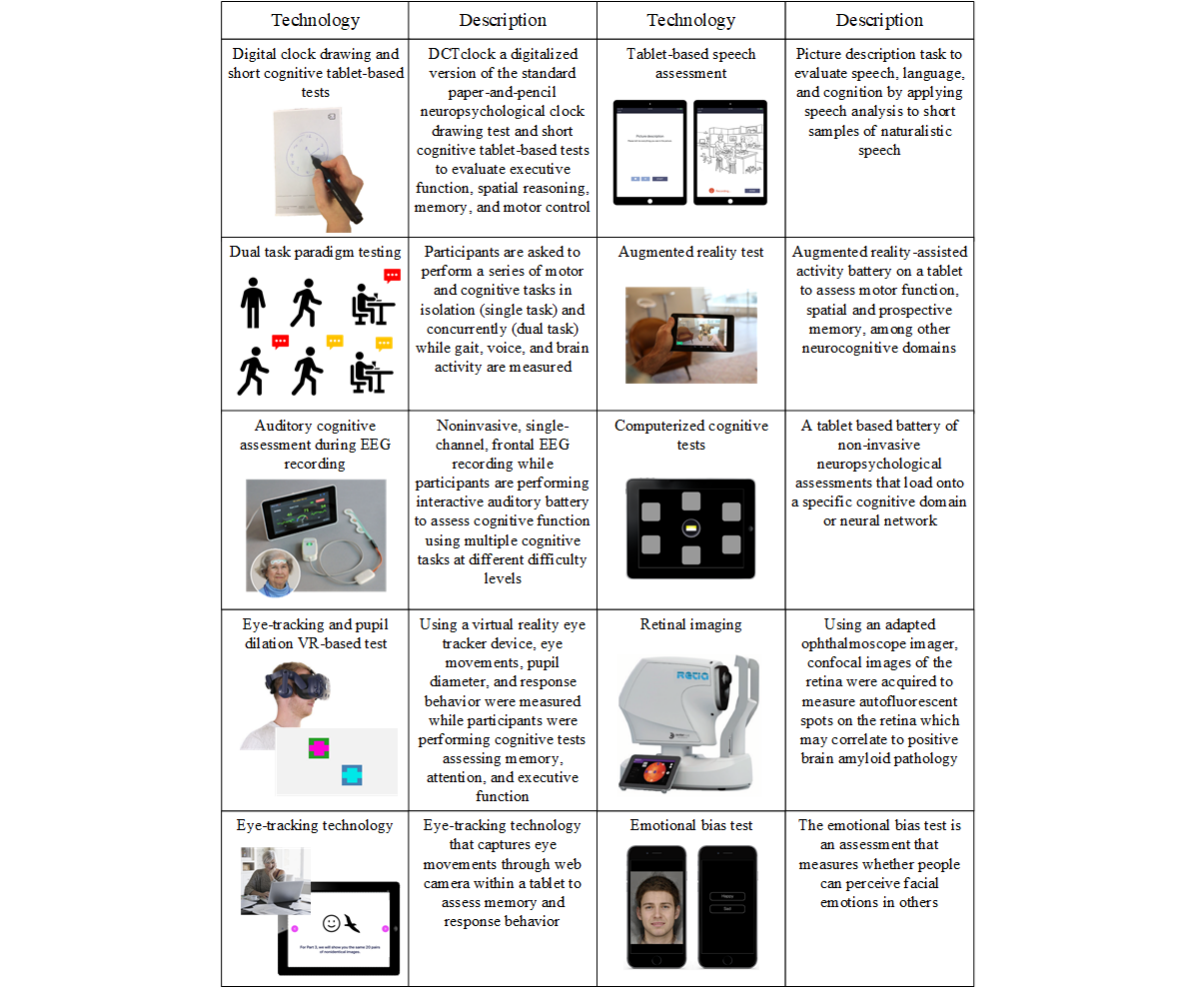
All technologies included in the MEDIA study. EEG: electroencephalogram; MEDIA: Method for Evaluating Digital Endpoints in Alzheimer Disease; VR: virtual reality.

Owing to the rapidly evolving field of digital technologies, it was considered beyond the scope of this study to ensure an exhaustive review and evaluation of all emerging digital end points and technology providers. Before starting the study, JC and KH reviewed and evaluated >100 companies to identify digital technologies that (1) augment conventional clinical assessments (using sensor technologies and machine learning), (2) allow direct physiological assessments of cognition (in particular, gait, eye movements, and brain activity), and (3) provide gamified cognitive assessments (such as AR, virtual reality, and computerized cognitive tests). Several potential technologies were identified in each category. Technology providers were prioritized if they had already demonstrated promising findings in the targeted patient population, showed promise during beta testing, or healthy volunteer testing. Figure 4 shows an overview of all digital end points selected for the MEDIA study.

#### AR, Spatial Navigation, and Memory Test (Digital Neuro Signature by Altoida)

Using Altoida’s technology, participants were asked to complete a battery of AR and motor activities on a tablet. First-time users can complete a brief training session to familiarize themselves with the activities and digital interface and ensure that they can successfully complete the different activity types. The testing session comprises the following activities: (1) motor activities where participants are asked to accurately trace colored paths on the screen with their index finger and to rapidly and accurately tap circles on the screen as they become highlighted; (2) an AR activity titled *Back in Time*, which primarily exercises spatial memory by asking the participant to place 3 AR objects in their environment and then locate the objects again in a similar fashion; and (3) an AR activity titled *Day Out*, which primarily measures prospective memory by asking the participant to learn a specific order of AR actions in an evacuation scenario. While performing 1 of the 2 AR activities described previously, the participants were asked to tap an on-screen icon when they hear a sound signal. The participant needs to discriminate between the high- and low-pitched sounds. This is a dual-task condition in which psychomotor processing speed is primarily assessed.

The data used for analysis is gathered by the sensors on the selected smart device (accelerometer and gyroscope) to determine parameters such as motion agility, speed, and smoothness of motion, as well as behavioral parameters such as recalled items placed in real space and the correct number of taps. These comprehensive parameters were used to calculate a Digital Neuro Signature score that can be used to predict an individual’s conversion from MCI to AD.

#### Computerized Cognitive Tests (Cambridge Neuropsychological Test Automated Battery by Cambridge Cognition)

The Cambridge Neuropsychological Test Automated Battery is a tablet-based battery of neuropsychological assessments, which load onto specific cognitive domains. The following test battery was chosen for this study: (1) Motor Screening Task, (2) Paired Associates Learning, and (3) Emotional Bias Task. The Motor Screening Task provides a general assay of whether sensorimotor or comprehension difficulties limit the collection of valid data from participants. Participants must touch the flashing cross, which is shown at different locations on the screen. The key outcome measure for this task is median latency. In Paired Associates Learning, which is a measure of episodic memory [25], boxes are opened on the screen to reveal a number of patterns. The participants are instructed to try to remember the location of each pattern. Each pattern is shown in the center of the screen in a randomized order, and the participant touches the box in which the pattern was located. The key outcome measures for this task are adjusted total errors and first attempt memory score. The Emotional Bias Task is an assessment of how people perceive facial emotions in others. The participant is required to view images of faces morphed between happy and sad emotions of varying intensities. They must then indicate whether they perceive the face shown on the screen as happy or sad. The key outcome measure for the Emotional Bias Task is the bias point, which is the proportion of trials selected as happy compared with the alternative emotion, adjusted to a scale of 0 to 15.

#### Instrumented Motor-Cognitive Dual Tasking (Physilog by GaitUp, NeuroVocalix by Cambridge Cognition, and Portable Electroencephalogram by Neurosteer)

**Figure 5:**
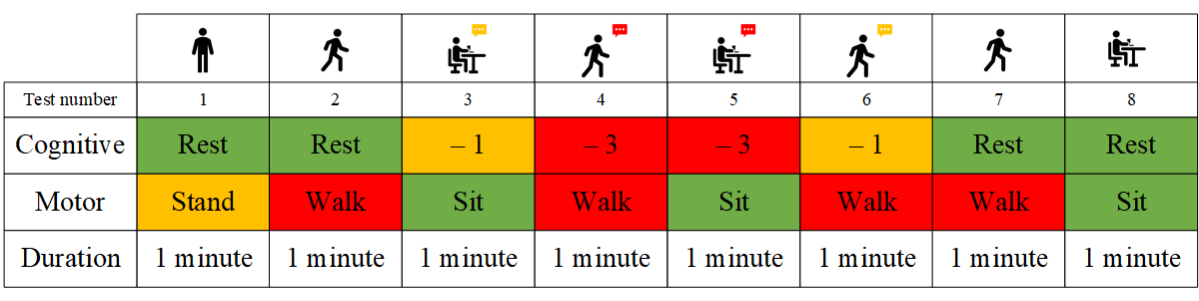
Task flow during dual tasking paradigm testing.

The participants were asked to perform a series of motor and cognitive tasks in isolation (single task) and concurrently (dual task). The motor task comprised walking at a self-selected pace for 1 minute. The cognitive task comprises counting backward in 2 difficulty levels (in steps of 1 and 3). The sequence and duration of the tasks are presented in Figure 5. To measure motor performance, 2 wearable inertial sensors (Physilog 5 by GaitUp) measuring acceleration (accelerometer; sampling frequency 128 Hz) and angular velocity (gyroscope; sampling frequency 128 Hz) were attached to participants’ feet. Using GaitUp’s algorithm, which has been validated in several patient populations [26-28], gait parameters such as but not limited to gait speed, step and stride length, step and stride time, and step and stride variability were extracted. To measure cognitive performance, a small microphone (Wireless GO; RØDE) was attached to participants’ clothes, which recorded the counting. Voice recordings were streamed to the NeuroVocalix web-based platform by Cambridge Cognition. The voice recordings will be analyzed for counting rate, number of errors, and correct counts, as well as vocal features during counting such as the length of pauses between numbers, energy, and pitch as features of the frequency spectrum. In addition, a wearable, single-channel electroencephalogram by Neurosteer was placed on the participants’ foreheads to measure brain function. The single- and dual-task phases will be used to compare the frontal brain activity between tasks and correlate to measures of cognitive load such as frequency-band power and Neurosteer’s brain activity biomarkers [29].

#### Neurosteer Auditory Cognitive Assessment

This test is a cognitive assessment based on auditory stimuli used to probe cognitive functionality. The testing includes auditory detection, n-back, auditory memory tasks, and resting state tasks. The detection and n-back tasks introduce different sequences of musical instrument melodies, eliciting participant responses. The detection task is used to test attention, inhibition response, and accuracy, whereas the n-back measures working memory. The memory tasks include statement recollection, mental clock imagery, and word recall. These are meant to test semantic memory, working memory, and memory consolidation and retrieval. Participants performed a 10-minute assessment while being recorded with a single-channel, medical-grade electroencephalogram. A total of 3 cognitive load levels (high, low, and rest) were used during the tasks and were correlated with behavioral performance and brain activity. The biomarkers extracted by the system were calculated using harmonic analysis and machine learning methods.

#### Winterlight Speech Assessment

Winterlight Speech Assessment was developed to record and analyze naturalistic speech using a tablet app. For the MEDIA study, each assessment included 2 picture description tasks in which participants were prompted to describe drawings of scenes presented on the tablet’s screen. Tasks of this type have been shown to be good proxies for spontaneous discourse and have been shown to be sensitive to speech changes in AD in previous studies [30-34]. The participant’s speech was recorded through the device’s microphone and analyzed via an automated speech analysis pipeline, generating variables reflecting different acoustic and linguistic properties of speech.

#### The Short-term Memory-Binding Test and Reading Task (by ViewMind)

Participants were asked to perform 2 cognitive tests, a short-term memory-binding test (STMBT) and a reading task while wearing a virtual reality headset with eye tracking (sampling rate of 120 Hz).

During the STMBT [35-37], participants were presented with a set of either 2- or 3-colored geometric shapes (Figure 2), depending on the cohort assignment at screening. The STMBT assesses the ability to temporarily hold bicolored objects whose colors have to be remembered either as individual features (baseline) or integrated within unified representations (binding).

The sentence corpus of the reading task comprised 40 regular sentences in Icelandic, which is the native language of all participants (eg, “Leifur heimsótti ættingja frá Evrópu í síðasta mánuði”; see the study by Fernández et al [38] for a description of a complete sentence corpus). The sentences comprised a well-balanced number of content and function words and had similar grammatical structure. Single sentences were presented at the centerline of the screen. The data used for analysis will be the x and y coordinates of eye movements together with time stamps and eye pupil diameter.

#### Retinal Imaging (Retia by NeuroVision)

Using an adapted ophthalmoscope (ophthalmoscope specification tailored for blue-light confocal autofluorescent imaging, with excitation illumination at 450 nm using a single flash light-emitting diode, emissions capture at ≥500 nm, and pixel resolution equivalent to 9 µm on the retina), confocal images of the retina were acquired at visit 2. After the administration of drops for eye pupil dilation (tropicamide 1% weight/volume) to the participant’s eyes to dilate the pupil to at least 3.5 mm in diameter, a series of autofluorescent images of the retina were acquired—nominally 18 images per eye. The entire noninvasive imaging procedure takes approximately 15 to 30 minutes. The participants’ raw image stack is processed by the automated software analysis package to assess the presence, size, position, shape, and other attributes of retinal autofluorescent spots. As the number of autofluorescent spots on the retina was correlated with the larger retinal amyloid burden in participants with AD versus controls [39,40], the likelihood of positive brain amyloid pathology, as determined by CSF sampling or amyloid PET imaging, will be calculated.

#### Digital Clock-Drawing and Cognitive Tablet-Based Drawing Tests (DCTclock by Linus Health)

##### DCTclock

DCTclock [41,42] is a digitized version of the standard rapid and noninvasive pen-and-paper neuropsychological clock-drawing test. The test involves participants drawing 2 clock faces on a piece of paper with a digital pen that precisely tracks and records the drawing behavior. The time-stamped positional data (x and y coordinates and time stamps) generated during this assessment are analyzed using proprietary machine learning algorithms that evaluate hundreds of features captured by the drawing process and the final output. By comparing test results with normative data, the system then determines whether the test is within normal limits and provides a detailed breakdown of performance on the various cognitive tasks evaluated during the test.

##### Cognitive Tablet-Based Drawing Tests

Participants were asked to complete a pretest assessment to familiarize themselves with the tablet and five short tablet-based drawing tests: (1) pretest, (2) pathfinding test, (3) symbol test, (4) trails test, and (5) tracing test. The pretest exercise involves copying waves. It was administered before completing the other tablet tests with the only goal of making the participant comfortable with drawing using the Apple Pencil and the iPad. In the pathfinding test, participants were asked to complete a series of mazes of increasing difficulty as quickly and accurately as possible. The symbol test comprises a key of 9 symbol-digit pairs followed by empty boxes with symbols on the top. Under each symbol, the participants must write down the corresponding symbol as fast as possible. In the trails test, the participant was instructed to connect a set of circles as quickly as possible, with the first part connecting numbers only and the second part connecting alternating numbers and letters. In the tracing test, the participant was prompted to trace a series of spirals and circles, first with their dominant hand and then with their nondominant hand. Expected features for further analysis are the time to finish a task, total strokes needed, and efficiency of drawing.

#### Imprint Assessment With Paired Recognition (Visual Paired Comparison by Neurotrack)

A tablet-integrated camera was used to record a video of the participant’s face while they were seated comfortably in a quiet, well-lit room in front of a tablet computer. Participants were shown a series of paired images during a familiarization phase and were then exposed to novel images. A second learning and test phase assessed paired associate learning and memory. Trial-level multimodal cognitive data (saccades, oscillations, gaze duration, and blinks), keystroke latency, performance accuracy, and discriminability for every participant on their digital multimodal cognitive tasks were collected. From these measures, measures of visual episodic memory, visual working memory, processing speed, executive function, and recognition discriminability will be derived.

### Participant Feedback Survey

A brief participant feedback survey was adapted from the Subject Usability Scale [43] and National Aeronautics and Space Administration Task Load Index [44] and was used in the MEDIA study to evaluate the participants’ experience and acceptance of the digital tools and assessments. The survey included 7 statements: “The device was easy to use”; “I needed to learn many things before I could get going with this device”; “This assessment was mentally demanding”; “This assessment was physically demanding”; “I enjoyed this assessment”; “I was insecure and/or frustrated during this assessment”; and “This assessment felt meaningful and relevant to difficulties I have in my daily life.” For these statements, participants indicated their degree of agreement or disagreement on a 6-point Likert scale ranging from 0 (strongly disagree) to 5 (strongly agree). There was an open question—“Is there anything else you would like to add?”—for open comments from the participants on the assessments.

### Statistical Analysis

As this is an exploratory study, the sample size was chosen pragmatically to balance statistical and feasibility considerations. Data from 12 participants per cohort were considered to provide sufficient information for the study objectives [45].

Descriptive statistics (ie, mean, median, range, and SD) of the total score from each conventional end point and questionnaire will be reported by the cohort. Estimates of the between-cohort standardized differences in the corresponding total scores will be provided.

Digital technologies will produce ≥1 outcome variable (features) for each participant and assessment time. On the basis of these features, psychometric properties will be assessed: acceptable range (ceiling and floor effects), reliability (test-retest variability), validity (correlation to conventional end points), and responsiveness (change from test and retest to challenge) as appropriate. Various statistical rules for classifying participants into cohorts will be explored. The goodness of these rules will be assessed by calculating the sensitivity, specificity, positive predictive value, and negative predictive value. Receiver operating characteristics curves will be constructed to determine cutoff points with the best trade-off between sensitivity and specificity. Data from the participant feedback survey will be summarized descriptively.

## Results

### Trial Status

**Figure 6:**
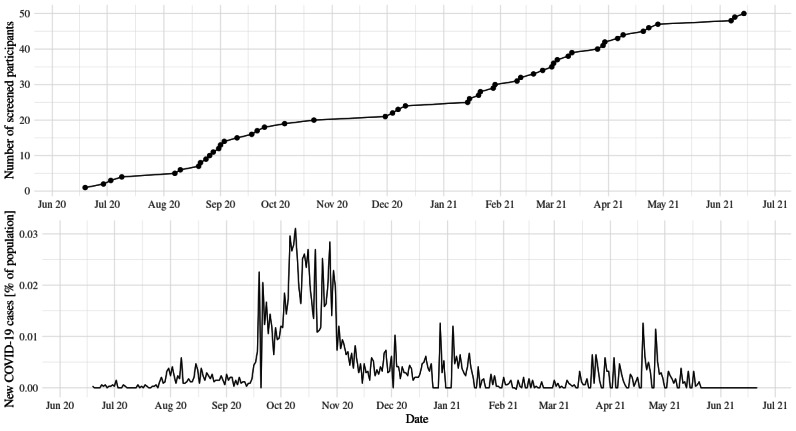
Study progress in Iceland during the worldwide COVID-19 pandemic.

Participant recruitment and data collection ran from June 2020 to June 2021. This study was fully conducted during the COVID-19 pandemic. Figure 6 shows the recruitment of the study participants together with daily new COVID-19 cases as a percentage of the population in Iceland (data from the study by Ritchie et al [46]).

### Trial Participants

The approximate average duration of all assessments at each study visit was as follows: visit 1, 1.48 (SD 0.25; range 1.10-2.17) hours; visit 2, 2.70 (SD 0.40; range 0.88-3.38) hours; visit 3, 2.17 (SD 0.33; range 1.47-2.90) hours; and visit 4, 1.63 (SD 0.28; range 0.83-2.18) hours. Table 1 shows demographic and conventional end point data. Medical history and comorbidities are listed in Multimedia Appendix 3.

**Table 1:** Demographics and baseline characteristics of the study population (N=50).

Demographics		Cohort 1: controls (n=13)	Cohort 2: presymptomatic (n=12)	Cohort 3: predementia (n=13)	Cohort 4: mild dementia (n=12)
**Age (years)**					
	Values, mean (SD)	68.1 (3.7)	72.4 (4.3)	70.6 (4.1)	69.3 (6.5)
	Values, median (range)	68 (63-73)	71 (65-78)	71 (62-78)	68 (61-80)
**Sex, n (%)**					
	Male	9 (69)	7 (58)	9 (69)	8 (67)
	Female	4 (31)	5 (42)	4 (31)	4 (33)
**Education, n (%)**					
	Higher education	9 (69)	4 (33)	4 (31)	3 (25)
	Upper secondary education	4 (31)	6 (50)	5 (39)	6 (50)
	Compulsory education	0 (0)	2 (17)	4 (31)	3 (25)
**BMI (kg/m^2^)**					
	Values, mean (SD)	26.7 (2.7)	25.3 (3.9)	26.6 (4.3)	26.7 (3.4)
	Values, median (range)	27.2 (22.5-31.4)	24.4 (20.5-34.1)	25 (21.1-33.5)	25.9 (23-35.4)
**MMSE^a^**					
	Values, mean (SD)	29.5 (1.1)	29.7 (0.7)	27.4 (2.2)	21.8 (1.5)
	Values, median (range)	30 (26-30)	30 (28-30)	28 (24-30)	22 (20-25)
**RBANS^b^**					
	Values, mean (SD)	101 (7)	98.4 (8.8)	75.7 (12.2)	66.3 (8.8)
	Values, median (range)	101 (88-115)	97 (84-118)	74 (49-95)	65 (55-83)
**CDR^c^ global**					
	Values, mean (SD)	0.04 (0.1)	0 (0)	0.5 (0)	0.7 (0.2)
	Values, median (range)	0 (0-0.5)	0 (0)	0.5 (0.5-0.5)	0.5 (0.5-1)
**CDR-SOB^d^**					
	Values, mean (SD)	0.1 (0.2)	0 (0)	1.7 (0.9)	3.5 (1.1)
	Values, median (range)	0 (0-0.5)	0 (0)	1.5 (0.5-3)	3.5 (1.5-5)
**ECog^e^ patient**					
	n-Nan^f^	1	0	1	0
	Values, mean (SD)	44.4 (4.7)	44.5 (4.5)	68.3 (28.5)	64.4 (10.2)
	Values, median (range)	44 (39-57)	44.5 (39-54)	56.5 (46-140)	67.5 (49-80)
**ECog caregiver**					
	Values, mean (SD)	45.5 (6.2)	47.5 (9.3)	67.6 (12.3)	88.7 (28.8)
	Values, median (range)	42 (39-56)	43 (39-65)	70 (53-91)	83 (53-137)
**NPI-Q^g^**					
	Values, mean (SD)	0.23 (0.6)	1.1 (2.2)	5.6 (5.3)	5.3 (6.9)
	Values, median (range)	0 (0-2)	0 (0-7)	4 (0-13)	3 (0-22)
**FACIT^h^**					
	n-Nan	0	0	1	1
	Values, mean (SD)	10 (2.3)	9.3 (3.7)	14.4 (7.8)	13.2 (5.4)
	Values, median (range)	11 (6.5-13)	8.75 (6-20)	11.2 (7-33)	11 (5.5-22)

^a^MMSE: Mini-Mental State Examination.

^b^RBANS: Repeatable Battery for the Assessment of Neuropsychological Status.

^c^CDR: Clinical Dementia Rating scale.

^d^CDR-SOB: CDR Sum of Boxes.

^e^ECog: Everyday Cognition.

^f^This is the number of missing values (eg, 1 study participant with a missing result for Everyday Cognition in cohort 1).

^g^NPI-Q: Neuropsychiatric Inventory–Questionnaire.

^h^FACIT: Functional Assessment of Chronic Illness Therapy.

## Discussion

### Expected Findings

The expected main findings of this study are as follows: (1) the accuracy of each of the 10 technologies in classifying participants into 4 cohorts according to the severity of cognitive impairment and dementia; (2) the psychometric properties of each of the digital technologies tested, including acceptable range (ceiling and floor effects), concurrent validity (correlation of ≥1 outcome measure of each of the technologies to traditional paper-and-pencil tests in AD), reliability (concordance of test and retest), and responsiveness (the sensitivity to change in a mild cognitive challenge model); and (3) feasibility of applying these technologies in drug trials based on study participant feedback.

Digital technologies hold promise in amending psychometric limitations associated with many conventional paper-and-pencil end points and transforming the way drug treatment effects are measured in clinical trials. For example, the digital pen used in this study to augment standard cognitive tests may reduce subjectivity and variability of well-established paper-and-pencil tests such as the clock-drawing test, trail-making test, or digit-symbol substitution test [42]; computerized testing implemented on tablets or smartphones with short cognitive test batteries may allow more frequent monitoring of cognition in the real world, thereby reducing variability and bias [47-49]; eye tracking while reading and voice analytics of conventional picture description tests may provide direct physiological assessments of cognition, which may increase sensitivity [30-37]; and AR or virtual reality testing may introduce more real-life assessments in a clinical setting [50,51]. More accurate and reliable efficacy end points could potentially reduce the number of failed or inconclusive trials and allow for more efficient drug development through shorter, smaller, less costly, and less burdensome drug trials, ultimately allowing more drugs to be studied and medicines to reach patients faster. However, it remains unclear how to best evaluate a wide range of novel and promising technologies, how to use them to derive efficacy end points, and how to advance them through early drug development processes.

The MEDIA study is an effort to establish a method for efficiently evaluating a range of technologies in a single study. Within the drug development framework, it is important to collect validation data and better understand operational feasibility before attempting to implement novel digital technologies in clinical drug trials. Clinical trials of drug interventions are highly complex and effortful undertakings and typically do not offer a good opportunity to study multiple novel digital end points. Often, these trials are already quite onerous for both participants and clinical sites, and the introduction of additional end points risks overburdening and jeopardizing the integrity of the primary objective of the study. Hence, carefully designed methodology studies such as the MEDIA study derisk drug intervention trials and offer valuable insights into the ceiling and floor effects, concurrent validity, reliability, and responsiveness of novel end points, as well as information about implementation, patient acceptance, and any operational complexity that a novel end point might add to a drug intervention trial.

A previous noninterventional study of 8 digital technologies for characterizing unipolar depression [52] successfully identified promising digital end points to advance as exploratory end points in numerous early phase clinical drug trials. However, this study lacked a challenge model and hence did not provide insights into sensitivity to change. A benign cognitive challenge model was implemented in the current MEDIA study, and the results will demonstrate if this is a feasible approach to study sensitivity to change in future methodological studies. The digital technologies included in the MEDIA study are in various stages of development. Some are already well established, with considerable validation data and clinical trial experience, whereas other technologies are still in early development. This will be taken into account when evaluating their performance.

Health authorities have published guidelines on the data required for a novel end point to be considered validated or qualified from a regulatory perspective [53-56]. Among the important qualities that an end point needs to meet are good psychometric properties (eg, lack of ceiling and floor effects), assay sensitivity (ie, sensitivity to drug treatment effects or disease progression), sufficient reliability and validity (eg, test-retest reliability and concurrent validity), and clinical meaningfulness. There are many ways of establishing clinical meaningfulness. This study used a participant feedback survey to understand patient acceptance of the end points and whether they considered the assessments meaningful to the problems they faced in their daily lives. Moreover, the outcomes of the novel digital end points will be correlated with the outcomes of conventional paper-and-pencil assessments of function, offering another method for assessing clinical meaningfulness. The topic of regulatory qualification of novel clinical end points is beyond the scope of this paper and will be addressed in a future publication.

Good concurrent validity occurs when a new end point demonstrates an appropriate correlation with an established gold standard. This is an important psychometric feature of sound clinical trial end points. However, as conventional neuroscience end points often display poor psychometric properties in various stages of AD (eg, ceiling effects in early predementia stages and floor effects in overt dementia), as well as rater errors and cultural bias, one might start to question the appropriateness of some of these conventional end points in establishing the concurrent validity of novel end points. Moreover, conventional end points are rarely pure measures of a single cognitive domain. For example, performing a memory task comprising a word list learning task relies not only on intact memory processes but also on attention, language comprehension, working memory, and executive function. Hence, it may be difficult to accurately interpret any potential lack of correlation between the conventional and novel cognitive end points. This dilemma will likely continue to challenge the development and validation of novel digital end points.

This study was conducted during the COVID-19 pandemic (SARS-CoV-2 virus pandemic). The full impact of the pandemic on this study is not clear [57]. First, the study was delayed at the beginning because of the implementation of preventive measures, and the overall duration of the project was prolonged because of various measures such as phases of lockdowns and quarantine. Originally, the planned duration of the study was approximately 6 months; however, this was prolonged to 1 year. Second, social distancing and infection control measures may have affected the digital assessments. For example, the use of face masks may have affected the quality of voice recordings and gait assessments. Finally, restrictions on social gatherings and regarding lockdown of commercial activities may have contributed to feelings of lethargy, anxiety, social isolation, disorientation to time, and a feeling of *every day is the same*. The psychological impact of the pandemic is yet to be further elucidated; however, it is clear that responses to questions about activities of daily living and neuropsychiatric symptoms during such unprecedented times are likely to be affected [58].

### Limitations

This exploratory methodology study has several limitations. Most notably, the small sample size necessitates findings to be confirmed in larger trials. Second, as this is a noninterventional study, it is difficult to establish sensitivity to change. One of the greatest risks that conventional end points pose to clinical drug development in neuroscience is the lack of sensitivity to changes and the possibility of missing important drug treatment effects. Hence, it is of utmost importance to demonstrate good assay sensitivity when studying novel digital end points to be implemented as efficacy measures in clinical trials. In this study, a benign cognitive challenge model was used in an attempt to assess the sensitivity to change of each digital end point. However, it is difficult to determine what challenge will be sufficient to reliably produce cognitive impairment in a cross-sectional study such as this one. Moreover, producing fatigue through late-night testing may not affect all cognitive domains to the same extent. Hence, the challenge model may affect participant performance on the various digital end points differently. In addition, as participants had no expectations of treatment benefits in this noninterventional study, it is possible that the digital end points would show different sensitivities to change in a clinical drug trial. Sensitivity to drug treatment effects cannot be fully evaluated until the digital end point is studied in a drug intervention trial. This is often the key validation data that novel digital end points are missing and is the most challenging data to acquire. This was a single-site study that provided limited information on how scalable the digital technologies are to multisite global trials and to what extent culture and language may be confounding factors. Finally, although the study included more men (33/50, 66%) than women (17/50, 34%), the proportions of men and women were, overall, similar across the 4 cohorts. It should also be noted that the education level was not evenly distributed across the cohorts; there was a greater number (and percentage) of participants with higher education in cohort 1 (9/13, 69%) than in cohort 2 (4/12, 33%), cohort 3 (4/13, 31%), and cohort 4 (3/12, 25%). As the education level may potentially be an important confounder, statistical analysis will be adjusted for it, as appropriate. Owing to the invasive procedures and sensitive biomarker information, there were no prospective CSF samples or PET imaging conducted in this noninterventional methodology study. Amyloid status was based on historical investigations that had to be aged <2 years. Historical reports were based on both CSF analysis (48/50, 96%) and PET imaging (2/50, 4%), and this study was not able to confirm any potential changes that may have occurred in amyloid status. Moreover, this study could not confirm any potential discrepancies between the 2 amyloid assessment procedures.

The findings of this study are planned to be disseminated in peer-reviewed journals and at key scientific conferences in 2022 and 2023.

### Conclusions

Although the surge of novel technologies is revolutionizing the way we approach cognitive testing, the challenge of systematically evaluating the performance of these digital end points remains. The MEDIA study delivers technology feasibility evaluations of 10 novel in-clinic digital end points and determines whether these tools provide tolerable and reliable measures of cognition, with improved psychometric properties and greater sensitivity and specificity than conventional clinical trial assessments. The MEDIA study psychometrically evaluates multiple digital cognitive end points head to head with conventional end points, as well as in a mild cognitive challenge model. Digital end point methodology studies such as the MEDIA study can efficiently avoid costly failures of improper digital end point implementation in clinical drug trials. Moreover, the MEDIA study may identify more sensitive screening tools for the earlier detection of AD, as well as potentially superior efficacy readouts for use in clinical drug development. A noninterventional study such as this is an important step toward establishing reliable and valid digital technologies ready to be used as efficacy end points in future clinical drug trials.

## Appendix

Multimedia Appendix 1 Inclusion and exclusion criteria.

Multimedia Appendix 2 Protocol deviations.

Multimedia Appendix 3 Medical history data.

## Abbreviations

AD: Alzheimer disease
AR: augmented reality
CDR: Clinical Dementia Rating scale
CDR-SOB: Clinical Dementia Rating scale Sum of Boxes
CSF: cerebrospinal fluid
ECog: Everyday Cognition scale
MCI: mild cognitive impairment
MEDIA: Method for Evaluating Digital Endpoints in Alzheimer Disease
NPI: Neuropsychiatric Inventory
NPI-Q: Neuropsychiatric Inventory–Questionnaire
PET: positron emission tomography
RBANS: Repeatable Battery for the Assessment of Neuropsychological Status
STMBT: short-term memory-binding test
