# Supplementary material for: Description of the Method for Evaluating Digital Endpoints in Alzheimer Disease Study: Protocol for an Exploratory, Cross-sectional Study
Source: JMIR Res Protoc. 2022 Aug 10;11(8):e35442. doi: 10.2196/35442 (PMC9403829; doi:10.2196/35442)
Supplement: Multimedia Appendix 1 [file resprot_v11i8e35442_app1.pdf]

Multimedia Appendix 1

Inclusion and exclusion criteria

Inclusion criteria

All Participants

1. Written informed consent must be obtained before any assessment is performed as part of the study.
2. Male or female, age 60 to 80 years inclusive, at the time of signing the informed consent.
3. Intellectually, visually and auditorily capable, fluent in, and able to read Icelandic, the language in which study assessments are administered (e.g., completion of at least six years of regular schooling or sustained employment or equivalent local level of knowledge). Additionally, all participants should have articulation and dominant limb ability to perform the cognitive tests.
4. In cases where data on amyloid status (elevated/not elevated) is not available or has not been collected, willingness to undergo brain amyloid assessments by CSF sampling via lumbar puncture or amyloid PET imaging prior to visit 2. The results from these assessments will classify participants as amyloid positive or amyloid negative prior to Visit 2.
5. Having a study partner who agrees to participate in the study and who is intellectually, visually, and auditorily capable, and fluent in, and able to read, the language in which study assessments are administered. Additionally, the study partner must be capable of and willing to:
    - Accompany the participant to visits that require the input of the study partner
    - Meet the definition of a “study partner” as described in Footnote 1.

For cognitively healthy amyloid negative and (pre-symptomatic) amyloid positive participants

1. Mini-Mental State Examination (MMSE) total score ≥ 24
2. Cognitively Healthy participants should be cognitively unimpaired as defined by:
    - Score of 85 or greater on the Repeatable Battery for the Assessment of Neuropsychological Status (RBANS) delayed memory index score AND
    - Clinical Dementia Rating (CDR) global score of 0, with two special exceptions:
        - If the RBANS delayed memory index score is between 70 and 84 (inclusive) AND the global CDR = 0, the participant may be allowed to continue ONLY if the investigator judges that cognition is unimpaired following review of the MCI/dementia criteria.
        - If the global CDR score = 0.5 AND the RBANS delayed memory index score is 85 or greater, the participant may be allowed to continue ONLY if the investigator judges that cognition is unimpaired following review of the MCI/dementia criteria.

- Previous RBANS or CDR results are valid for this study if performed by the site within 6 months and no objective evidence or subjective reports of worsening cognition

3. For participants who have previously undergone brain amyloid assessments:
   - Cognitively healthy (pre-symptomatic) amyloid positive participants: Amyloid positive PET scan or CSF screening investigations for evidence of elevated brain amyloid can be waived if results are available from a previous amyloid PET scan or CSF lumbar puncture.
     In cases where both CSF Aβ and amyloid PET imaging test results are available, at least one should be indicative of elevated brain amyloid. Cut-off levels for amyloid positivity and Aβ-42 and/or Aβ-Tau ratios will be based on site-specific criteria.

Cognitively healthy amyloid negative participants: Amyloid negative PET scan or CSF screening investigations can be waived if results, no older than 12 months, are available from a previous amyloid PET scan or CSF lumbar puncture.
In cases where both CSF Aβ and amyloid PET imaging test results are available, both should be indicative of non-elevated brain amyloid. Cut-off levels for amyloid positivity and Aβ-42 and/or Aβ-Tau ratios will be based on site-specific criteria.

For MCI and mild AD participants

1. Mini-Mental State Examination (MMSE) total score ≥ 20.
2. Elevated brain amyloid (as measured by CSF Aβ or amyloid PET imaging). Amyloid positive PET scan or CSF screening investigations for evidence of elevated brain amyloid can be waived if results are available from a previous amyloid PET scan or CSF lumbar puncture.

   In cases where both CSF Aβ and amyloid PET imaging tests are performed, at least one should be indicative of elevated brain amyloid. Cut-off levels for amyloid positivity and Aβ-42 and/or Aβ-Tau ratios will be based on site specific criteria.
3. For a diagnosis of mild AD, participants have to meet the NIA-AA criteria for probable AD
4. For a diagnosis of MCI, participants have to meet NIA-AA criteria for MCI due to AD. Taking a cholinesterase inhibitor for a minimum period of 3 months prior to the day of inclusion into the study or not have been taking a cholinesterase inhibitor for a minimum period of 3 months prior to the day of inclusion into the study.

Footnote 1: The study partner is expected to spend sufficient time with the participant to be familiar with his/her overall function and behavior, and be able to provide adequate information about the participant including: (a) knowledge of domestic activities, hobbies, routines, social skills and basic activities of daily life; (b) work and educational history; (c) cognitive performance, including memory abilities, language abilities, temporal and spatial orientation, judgment, and problem solving; (d) emotional and psychological state; and (e) general health status. Although the study partner is expected to accompany the participant to all relevant visits, if unavailable to attend a given site visit (e.g., if traveling or sick), his/her input to clinical scales can be organized via a telephone interview for NPI-Q. Yet for CDR and ECog, a site visit is preferable. The study partner and/or the participant will be requested to call the site to inform them about any study partner unavailability or transition to a new one.

Exclusion criteria

Participants fulfilling any of the following criteria are not eligible for inclusion in this study:

1. Current medical or neurological condition other than Alzheimer's disease that might impact cognition or performance on cognitive assessments, e.g., Huntington's disease, Parkinson's disease, Lyme disease, syphilis, schizophrenia, bipolar disorder, major depressive disorder (MDD), suicidal ideation, active seizure disorder, current alcohol/drug abuse or dependence, previous head injuries with loss of consciousness, or any other condition that might impact cognition or performance on cognitive assessments as judged by the investigator.
2. Advanced, severe progressive or unstable disease that may interfere with the safety, tolerability and study assessments, or put the participant at special risk as judged by the investigator
3. Any concomitant cancer treatment.
4. Use of other investigational drugs prior to screening until:
   - Small molecules: after five half-lives, or within 30 days until the expected pharmacodynamic effect has returned to baseline, whichever is longer.
   - Biologicals: blood concentration has returned to baseline (or below serological responder threshold) for antibodies induced by active immunotherapy; or five half-lives for monoclonal antibodies or other biologicals.
5. Use of illicit drug and alcohol use 48 hours prior to clinic visits.
6. Previous brain MRI results showing findings unrelated to AD that, in the opinion of the investigator might be a leading cause of cognitive decline, or might pose a risk to the participant (e.g., extensive white matter lesions, stroke, cerebrovascular disease as evidenced by multiple lacunar infarcts ≤ 20 mm or single infarct > 20 mm, evidence of cerebral contusion, encephalomalacia, aneurysms, vascular malformation, subdural hematoma or space-occupying lesions).
7. If CSF sampling is scheduled for this participant: Contraindication to LP, e.g., low platelet count, abnormal prothrombin time international normalized ratio (PTINR), history of back surgery (with the exception of microdiscectomy or laminectomy over one level), signs or symptoms of intracranial pressure, spinal deformities or other spinal conditions that in the judgment of the investigator would preclude a LP.

   If PET scans are scheduled for this participant: Total dosimetry above the acceptable exposure in the country when combining the previous or planned Nuclear Medicine Radiology exposure and the scheduled study PET scan(s).

Currently enrolled in the treatment period of any other clinical trial involving an investigational product or any other type of medical research judged not to be scientifically or medically compatible with this study.
