# Supplementary material for: Description of the Method for Evaluating Digital Endpoints in Alzheimer Disease Study: Protocol for an Exploratory, Cross-sectional Study
Source: JMIR Res Protoc. 2022 Aug 10;11(8):e35442. doi: 10.2196/35442 (PMC9403829; doi:10.2196/35442)
Supplement: Multimedia Appendix 2 [file resprot_v11i8e35442_app2.pdf]

Multimedia Appendix 2

Protocol Deviations (PDs)

There were 4 important PDs reported. 2 PDs were related to changes in assessment/procedure because of COVID-19 pandemic and 2 PDs were related to inclusion criteria. Please find additional details below:

- Assessment/procedure changed due to COVID-19: one participant received a text message during Visit 3, asking him/her to go into quarantine. The rest of the visit was postponed.
- Assessment/procedure changed due to COVID-19: one participant didn't have Visit 4 performed on the same day as Visit 3.
- IC#3.5: one participant had only been taking a cholinesterase inhibitor for approximately 2 months at the time of screening and continued participation in study without being re-screened. Participant was not discontinued to the study as no safety issues were identified. In addition, repeating conventional assessments may have produced learning effects that could have impacted study integrity more than the lack of 3-month stable dementia medications and finally although 3 months stable dose was our inclusion criteria, in reality the difference of 2 or 3 months may not be significant.
- IC#1.2: one participant was randomized, despite being over the age of 80 and was then discontinued after Visit 1.

In addition, there were 7 non-important PDs related to out of window visits (ranging from 3 to 36 days).
