# Supplementary material for: Description of the Method for Evaluating Digital Endpoints in Alzheimer Disease Study: Protocol for an Exploratory, Cross-sectional Study
Source: JMIR Res Protoc. 2022 Aug 10;11(8):e35442. doi: 10.2196/35442 (PMC9403829; doi:10.2196/35442)
Supplement: Multimedia Appendix 3 [file resprot_v11i8e35442_app3.pdf]

Multimedia Appendix 3

Medical history data. Average number of comorbidities per participant = 3.64 (SD 1.8)

Comorbidity	N° participants (%)	Examples
Vascular disorders	24 (54.5%)	Hypertension, atherosclerosis
Nervous system disorders	21 (46.7%)	Alzheimer's disease, restless leg syndrome, transient ischemic attack
Psychiatric disorders	21 (47.7%)	Anxiety, depression, insomnia
Metabolism and nutrition disorders	18 (40.9%)	B12 deficiency, hypercholesterolemia, diabetes
Gastrointestinal disorders	10 (22.7%)	Reflux esophagitis, diaphragmatic hernia
Musculoskeletal and connective tissue disorders	10 (22.7%)	Gonarthrosis, hip arthrosis, polymyalgia
Cardiac disorders	10 (22.7%)	Coronary heart disease, ischemic heart disease, supraventricular tachycardia
Reproductive system and breast disorders	8 (18.2%)	Prostatic hypertrophy, vulvovaginal atrophy
Surgical and medical procedures	7 (15.9%)	Nephrectomy, abdominoplasty, hysterectomy
Endocrine disorders	5 (11.4%)	Hypothyroidism, hypotestosteronemia
Neoplasms benign, malignant and unspecified (incl. cysts and polyps)	5 (11.4%)	MGUS, prostate cancer
Respiratory, thoracic and mediastinal disorders	5 (11.4%)	Obstructive sleep apnea syndrome, lung disease obstructive
Renal and urinary disorders	3 (6.8%)	Kidney failure, neurogenic bladder
No comorbidities	3 (6.8%)	
Eye disorders	3 (6.8%)	Glaucoma, cataracts, macular degeneration
Immune system disorders	2 (4.5%)	Pollen allergy
Skin and subcutaneous tissue disorders	2 (4.5%)	Psoriasis, alopecia areata
Investigations	1 (2.3%)	High cholesterol
General disorders and administration site conditions	1 (2.3%)	Ankle oedema
Ear and labyrinth disorders	1 (2.3%)	Benign positional vertigo
